# A Genome-Wide Screen for the Exonisation of Reference SINE-VNTR-Alus and Their Expression in CNS Tissues of Individuals with Amyotrophic Lateral Sclerosis

**DOI:** 10.3390/ijms241411548

**Published:** 2023-07-17

**Authors:** Abigail L. Pfaff, Vivien J. Bubb, John P. Quinn, Sulev Koks

**Affiliations:** 1 Perron Institute for Neurological and Translational Science, Perth, WA 6009, Australia; 2 Centre for Molecular Medicine and Innovative Therapeutics, Murdoch University, Perth, WA 6150, Australia; 3 Department of Pharmacology and Therapeutics, Institute of Systems, Molecular and Integrative Biology, University of Liverpool, Liverpool L69 3BX, UK

**Keywords:** SINE-VNTR-Alu, retrotransposon, amyotrophic lateral sclerosis, exonisation, gene expression

## Abstract

The hominid-specific retrotransposon SINE-VNTR-Alu (SVA) is a composite element that has contributed to the genetic variation between individuals and influenced genomic structure and function. SVAs are involved in modulating gene expression and splicing patterns, altering mRNA levels and sequences, and have been associated with the development of disease. We evaluated the genome-wide effects of SVAs present in the reference genome on transcript sequence and expression in the CNS of individuals with and without the neurodegenerative disorder Amyotrophic Lateral Sclerosis (ALS). This study identified SVAs in the exons of 179 known transcripts, several of which were expressed in a tissue-specific manner, as well as 92 novel exonisation events occurring in the motor cortex. An analysis of 65 reference genome SVAs polymorphic for their presence/absence in the ALS consortium cohort did not identify any elements that were significantly associated with disease status, age at onset, and survival. However, there were transcripts, such as transferrin and *HLA-A*, that were differentially expressed between those with or without disease, and expression levels were associated with the genotype of proximal SVAs. This study demonstrates the functional consequences of several SVA elements altering mRNA splicing patterns and expression levels in tissues of the CNS.

## 1. Introduction

**Figure 1:**
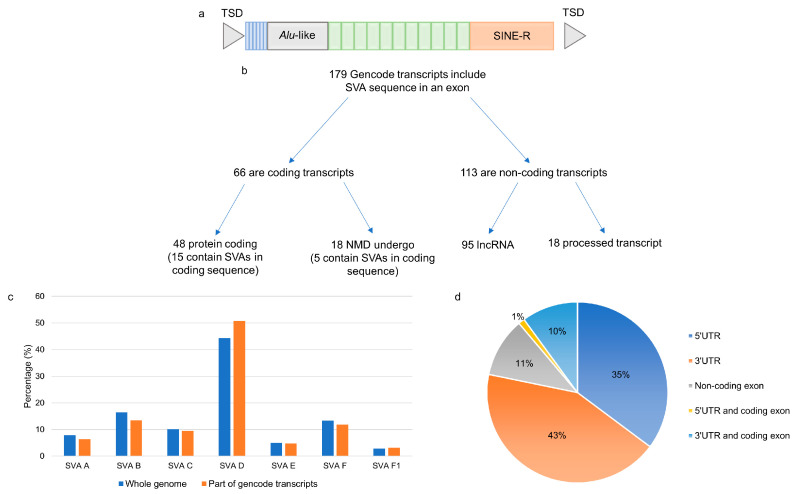
Reference SVAs were included in exons of annotated transcripts from Gencode. (**a**) The structure of a full-length SVA consisting of a CCCTCT-repeat variable in length, an *Alu*-like sequence, a GC-rich VNTR, a SINE region, and a poly A-tail flanked by target site duplications. (**b**) Sequence from 126 reference SVAs were included as exons in 179 different transcripts in the Gencode database. (**c**) The proportion of each SVA subtype that was part of a transcript was similar to the overall proportion of each subtype across the genome. (**d**) The location of the SVA sequence in the transcript structure and the majority of the SVAs formed part of the 5′ and 3′ UTRs of the transcripts. TSD–target site duplication, SINE-R–short interspersed element region, UTR–untranslated region.

SINE-VNTR-Alus (SVAs) are the youngest family of retrotransposons in the human genome and are so named for their different domains [1]. SVAs, from 5′ to 3′, consist of a hexamer repeat, an *Alu*-like sequence, a GC-rich variable number tandem repeat (VNTR), a short interspersed element region (SINE-R), and a poly A-tail (Figure 1a). Retrotransposons, including SVAs, have had a significant impact on the human genome, affecting genomic function through the introduction of novel regulatory sequences. These sequences include promoters and transcription factor binding sites, cause transcriptional interference and altered methylation patterns, and are incorporated into the mRNA of existing genes [2]. Retrotransposition of SVA elements requires the proteins encoded by the long interspersed element-1 (LINE-1) [3] and occurs in approximately 1/63 live births [4]. This ongoing mobilisation of SVAs has contributed to genetic variation between populations and individuals [5], and insertion polymorphisms have been associated with the regulation of gene expression and disease development and progression [6,7,8].

Retrotransposon insertion polymorphisms (RIPs) have been identified as expression quantitative trait loci (eQTLs), uncovering elements whose genotypes were associated with differential gene expression that can be both population- and tissue-specific [8,9,10,11]. Investigating these regulatory effects led to the identification of candidate RIPsthat could lead to disease through their influence on gene expression. For example, SVA insertion into an enhancer of the *B4GALT1* gene, a region implicated in inflammatory conditions and autoimmune diseases, was associated with a reduction in the expression of the gene in B-cells that would lead to a pro-inflammatory state [8]. A recent study evaluating the role of structural variants in gene regulation in the brain showed that SVA elements were enriched not only as eQTLs but also as splice and protein QTLs as well [10].

To date, at least 26 SVAs have been identified as the genetic cause of diseases such as cancer predisposition syndromes, X-linked dystonia parkinsonism, antithrombin deficiency, and spinal muscular atrophy [12,13,14]. More than half of these insertions caused aberrant splicing patterns through mechanisms such as exon skipping, exonisation events (which introduced premature stop codons), or the activation of cryptic splice sites. These rare SVA insertions have robust effects, causing disease phenotypes; however, the SVAs either fixed in the human genome or common RIPs could also influence splicing patterns, exerting more subtle effects on cellular function. The SVA-containing transcripts may become more predominantly expressed over time if it is not detrimental to cellular health, providing a mechanism through which novel gene sequences and functions can be acquired. The inclusion of an intronic element derived from a retrotransposon event into a gene transcript, also known as exonisation, has contributed to the evolution of the human genome, and studies have mainly focussed on *Alu* elements due to the frequency of their occurrence [15].

The aim of our study was to investigate the influence of reference genome SVAs on splicing patterns and gene expression in tissues of the CNS, with a specific focus on individuals with the neurodegenerative disease Amyotrophic Lateral Sclerosis (ALS). ALS is a progressive neurological disorder that results in the degeneration of both upper and lower motor neurons, with death occurring within 3–5 years of symptom onset. The exact aetiology of motor neuron degeneration is still to be characterised; however, investigations of genetic variation and transcriptomic changes have identified multiple genes and pathways involved in disease risk and development [16,17]. SVAs are known to affect gene expression and contribute to genetic diversity; therefore, we utilised a combination of data types, specifically whole genome and RNA sequencing data from the ALS consortium of the New York Genome Center (NYGC) alongside DNA and RNA samples from the NIH NeuroBioBank, to study their role in ALS. Using this approach, we identified transcript expression and splicing modulated by SVA variation and the differential expression of SVA-containing transcripts between tissues and individuals with ALS and those without the disease.

## 2. Results

### 2.1. Exonisation of Reference SVAs in Transcripts That are Differentially Expressed in CNS Tissues

**Figure 2:**
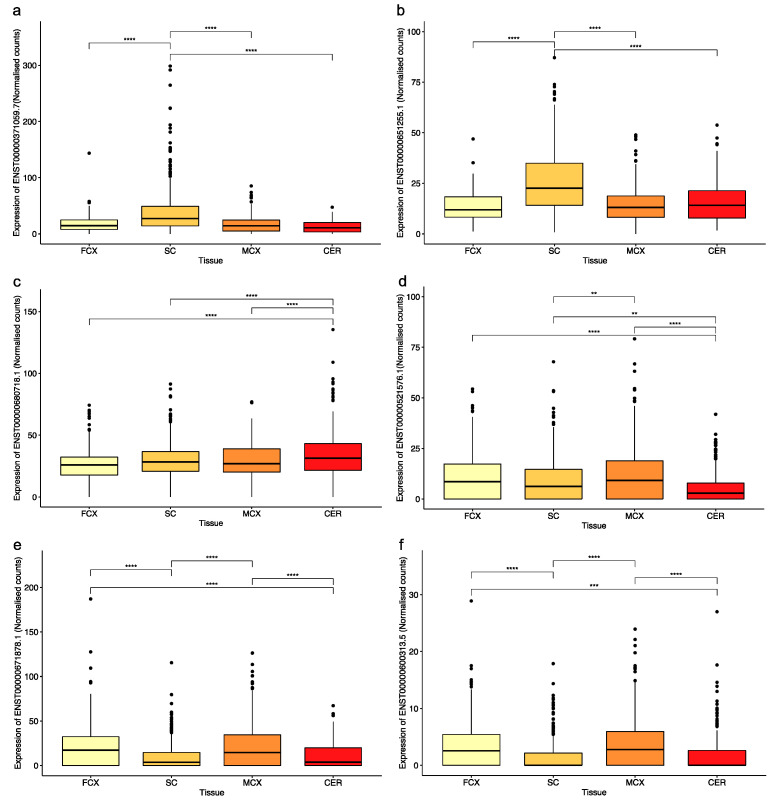
There was a significant difference in the expression of the transcripts with coding exons that contained SVA sequences between tissues of the CNS. The graphs show normalised counts across four tissues from the Target ALS cohort for transcripts from the following genes: (**a**) LEPR, (**b**) *MS4A4A*, (**c**) *C2CD3*, (**d**) *HGSNAT*, (**e**) *ALDH3A2*, and (**f**) *ZNF675*. The number of samples analysed per tissue were as follows (includes data from both NNC and individuals with ALS or ALSND): FCX–172, SC–383, MCX–317, and CER–187. An ANOVA test with a Tukey adjustment for pairwise comparisons was performed. ** *p* < 0.01, *** *p* < 0.001, **** *p* < 0.0001. FCX–frontal cortex, SC–spinal cord (includes data from cervical, thoracic, and lumbar regions), MCX–motor cortex (medial and lateral regions), and CER–cerebellum.

A genome-wide analysis of 2662 reference SVAs in relation to exons of gene transcripts in the Gencode database (V39) identified 179 different transcripts containing sequences from 126 SVAs (Figure 1b). Of the 179 transcripts, 66 were coding, 48 of which encoded proteins, and 18 were predicted to undergo nonsense-mediated decay. There were 113 non-coding transcripts containing SVA sequences, 95 of which were long non-coding RNAs (lncRNAs), and 18 were classified as processed transcripts (Supplementary Materials Table S1). There was no preference for a particular SVA subtype to be incorporated into a transcript (Figure 1c). However, there was a preference for those SVAs in the same orientation as the transcript to contribute to exonic sequences compared to those in the opposite orientation. Of the 2662 reference SVAs in the human genome, 58% were in introns, 29% of which were in the same orientation as the gene transcript in which they had inserted. This is significantly lower than the 82% of SVAs contributing to exonic sequences that are in the same orientation as the transcript (*p* > 0.00001; Fisher’s exact test). This is in concordance with the underrepresentation of SVA on the coding strand, which has been reported previously [18]. The majority of the 126 SVAs identified as part of a gene transcript contributed to sequences of the 5′ and 3′ UTRs of the transcripts in which they were located; however, several SVAs formed part of the coding sequence (Figure 1d). There were 15 transcripts where the SVA encoded part of the protein, and their expression levels were assessed in four tissues of the CNS in the Target ALS cohort. In 13 of the 15 transcripts, the SVA contributed to the last coding exon, and the 3′UTR and the remaining 2 contributed to the first coding exon and the 5′UTR. The Target ALS RNA sequencing data analysed included individuals diagnosed with ALS spectrum MND (ALS), ALS with other neurological disorder (ALSND) and non-neurological controls (NNC), and four tissues: motor cortex (MCX), frontal cortex (FCX), spinal cord (SC), and the cerebellum (CER). The expression of the 15 transcripts partly encoded by an SVA was detected across the CNS albeit at different levels, and some were only detected with a low number of reads (Figure 2 and Supplementary Materials Figure S1). Eleven of the transcripts were differentially expressed between at least two tissues of the CNS analysed (Figure 2 and Supplementary Materials Figure S1). The expression levels of the 15 transcripts were compared between ALS/ALSND and NNC in the four tissues. There were three transcripts—ENST00000651255.1 (*p* = 0.0008), ENST00000600313.5 (*p* = 0.007), and ENST00000521576.1 (*p* = 0.02)—that were differentially expressed in the spinal cord, frontal cortex, and cerebellum, respectively.

### 2.2. Identification of Novel Exonised Reference SVAs in the Motor Cortex

The initial analysis of reference SVAs identified those that were part of annotated transcripts, and we extended this to identify the SVA exonisation events not reported in the Gencode database. By overlapping the coordinates of exon junctions from 10 of the motor cortex samples from the NIH NeuroBioBank (five individuals with ALS and five controls), 92 potential novel exonised SVAs were identified (Supplementary Materials Table S2). These were defined as an exon junction from within an SVA into an adjacent exon that was part of a known transcript. On average, a novel exonisation event was detected 2.6 times; 50 novel exonised SVAs were only detected in a single individual, whereas there was evidence for 4 of the novel exonised SVAs in all 10 samples analysed (Supplementary Materials Table S2). The majority of the SVAs, 67/92 (73%), were located in the same orientation as the transcript. This was similar to the SVAs that were already annotated as forming part of a Gencode transcript (82%) and, in contrast to all reference SVAs, located in introns (29%).

**Figure 3:**
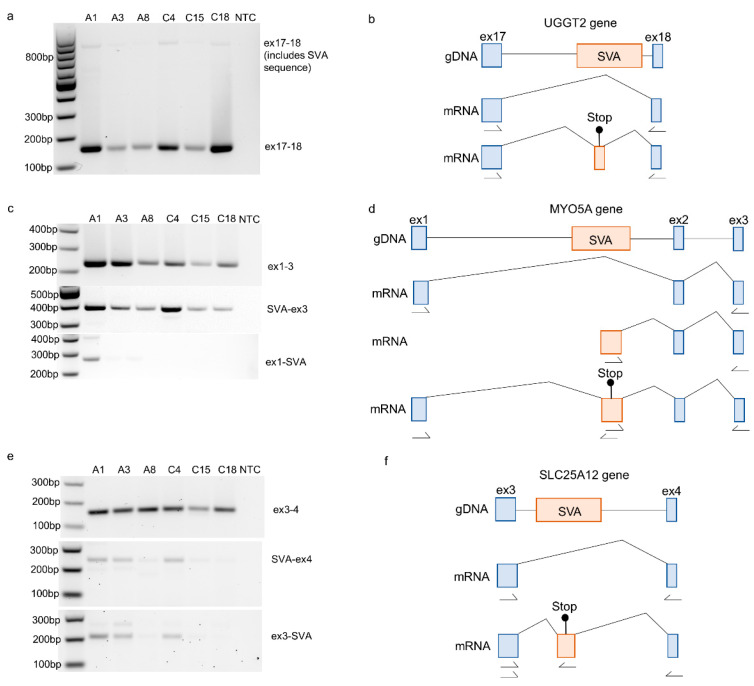
Validation of novel exonised SVAs using RT-PCR identified in RNA-sequencing data from the motor cortex. (**a**) Amplification of exon 17 to 18 of the *UGGT2* gene (exons numbered according to ENST00000376747.8). The PCR product representing the Gencode-reported transcript was 159 bp, and the inclusion of the SVA sequence increased the size to approximately 813 bp. (**b**) Schematic of the *UGGT2* gDNA and the mRNA of the two transcripts detected; according to the RNA sequencing junction data, the included sequence of the SVA would introduce a stop codon. (**c**) Amplification of exons 1 to 3 of the *MYO5A* gene (exons numbered according to ENST00000356338.11). The PCR product representing the Gencode-reported transcript was 229bp. The SVA-containing transcripts were amplified using primers located within the SVA up- and downstream exons, and the PCR products were expected to be 366 bp for SVA to exon 3 and 259 bp from exon 1 to SVA. (**d**) Schematic of the *MYO5A* gDNA and the mRNA of the three transcripts detected (the canonical transcript, a transcript initiating with the SVA sequence, and a transcript including the SVA sequence between exon 1 and 2). According to the RNA sequencing junction data, the included sequence of the SVA would introduce a stop codon. (**e**) Amplification of exon 3 to 4 of the *SLC25A12* gene (exons numbered according to ENST00000422440.7). The PCR product representing the Gencode-reported transcript was 159bp. The SVA-containing transcripts were amplified using primers located within the SVA up- and downstream exons, and the PCR products were expected to be 238 bp for SVA to exon 4 and 206 bp from exon 3 to SVA. (**f**) Schematic of the *SLC25A12* gDNA and the mRNA of the three transcripts detected (the canonical transcript and a transcript including the SVA sequence between exons 3 and 4). According to the RNA sequencing junction data, the included sequence of the SVA would introduce a stop codon. Location of primers are indicated by arrows.

To validate the exonised SVAs identified in the RNA sequencing data, primers were designed to amplify the transcripts from the three genes containing SVA sequences using RT-PCR and RNA from the NIH NeuroBioBank samples. The amplification of exons 17–18 of the *UGGT2* gene (ENST00000376747.8) in the MCX RNA detected the expression of the annotated Gencode transcript alongside a larger product corresponding to the inclusion of part of the SVA located in intron 17 (Figure 3a,b). An SVA located in intron 1 of the *MYO5A* gene (ENST00000356338.11) was shown to form junctions with exons 1 and 2 of the gene. Validation using primers located within exon 1, the SVA itself, and exon 3 demonstrated the existence of two additional transcripts: one originating in the SVA and another that included part of the SVA sequence between exons 1 and 2 (Figure 3c,d). This is consistent with the SVA acting as an alternative start site for the *MYO5A* gene. The third novel exonisation event to be validated was that of an SVA located within intron 3 of the *SLC25A12* gene (ENST00000422440.7). The amplification of MCX RNA detected the expression of a transcript that included sequences from the SVA as well as the expression of the annotated Gencode transcript (Figure 3e,f). An examination of the RNA sequencing data in all three examples (shown in Figure 3) determined that the SVA sequence included between the two exons of the existing transcript would introduce additional stop codons.

### 2.3. A Polymorphic SVA in an Intron of the CASP8 Gene Alters Transcript Sequence and Expression Levels

**Figure 4:**
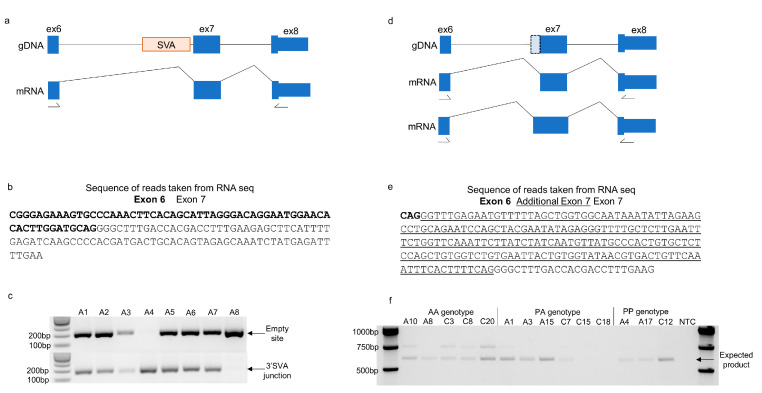
The absence of the *CASP8* SVA results in the expression of an additional transcript containing an intronic sequence. (**a**) Schematic showing the location of the SVA in the *CASP8* gene gDNA (exons represented by blue boxes numbered according to transcript ENST00000323492.11) and the mRNA expressed from this transcript according to the Gencode database. (**b**) Sequence of reads originating from exons 6 and 7 taken from the MCX RNA sequencing bam files that correspond with the transcripts represented in the Gencode database. (**c**) Representative image of the different presence/absence genotypes of the *CASP8* SVA from eight individuals in the NIH NeuroBioBank cohort. A 204 bp PCR product over the empty site (absent allele) and a 168 bp PCR product over 3′ junction of the SVA (present allele) were amplified from gDNA to determine the genotype of each individual in the 40 samples from the NIH NeuroBioBank cohort. (**d**) Schematic of the *CASP8* gene gDNA without the SVA, the mRNA expressed according to Gencode, and the previously unreported transcript containing an intronic sequence adjacent to exon 7 (numbered according ENST00000323492.11), the latter of which is represented by the dotted line in the gDNA. (**e**) Sequence of reads originating from exons 6 and 7 taken from the MCX RNA sequencing bam files that included an additional 191 bp of sequence from the intervening intron adjacent to exon 7. (**f**) Amplification of *CASP8* transcripts expressed in the MCX of selected individuals from the NIH NeuroBioBank cohort with primers located in exons 6 and 8 as numbered in parts (**a**,**d**). The PCR product expected for the transcript present in Gencode is 600 bp in size (indicated by the arrow) and was detected in most of the individuals shown. The larger PCR product containing an additional sequence was only detected in those who are homozygous absent for the SVA in *CASP8*. The locations of primers are indicated by arrows.

To investigate the influence of SVAs on splicing events further, we focussed on 27 intronic SVAs that were within 100 bp of an exon. Many of the determinants of alternative splicing are located in close proximity to the intron–exon boundaries [19], and *Alu* retrotransposons have been shown to influence splicing when located within 100 bp of an exon [20]. The exon junctions from the NIH NeuroBioBank MCX RNA sequencing data near the 27 SVAs were inspected. This identified two genes—*CACNB4* and *CASP8*—that contained exon junctions adjacent to the SVA and not part of the annotated transcripts. The SVA located in intron 6 of *CASP8* was within 100 bp 5′ of exon 7 (numbered according to transcript ENST00000323492.11) (Figure 4a) and has previously been reported as polymorphic for its presence/absence in the genome and associated with intron retention [21]. The read sequences in this region were extracted from the MCX RNA sequencing data corresponding to the annotated junction (exon 6–7) (Figure 4b) and a second junction that included a sequence from exons 6 and exon 7 (intronic sequence 5′ and 3′ of the SVA) (Figure 4e). The NIH NeuroBioBank samples were genotyped for the presence/absence of the *CASP8* SVA (Figure 4c). The annotated junction (exon 6–7) was present in all 39 NIH NeuroBioBank MCX RNA sequencing samples, whereas the novel junction was only present in individuals with at least one absent allele of the SVA. This is consistent with the model in which the absence of the SVA results in an alternative splice acceptor site in intron 6 being used and a larger exon 7 (Figure 4d,e). This was further supported by the presence of a larger PCR product in the homozygous absent individuals of the NIH NeuroBioBank cohort when using RT-PCR to amplify exons 6–8 of the *CASP8* transcript. In alignment with this, the 600 bp product corresponding to the annotated transcript was detected in individuals of all three genotypes (homozygous absent-AA, heterozygous-PA, and homozygous present-PP) (Figure 4f). There were 15 different transcripts of the *CASP8* gene that include the two exons flanking the SVA insertion; therefore, it is difficult to predict the effects of the alternative splice acceptor site.

**Figure 5:**
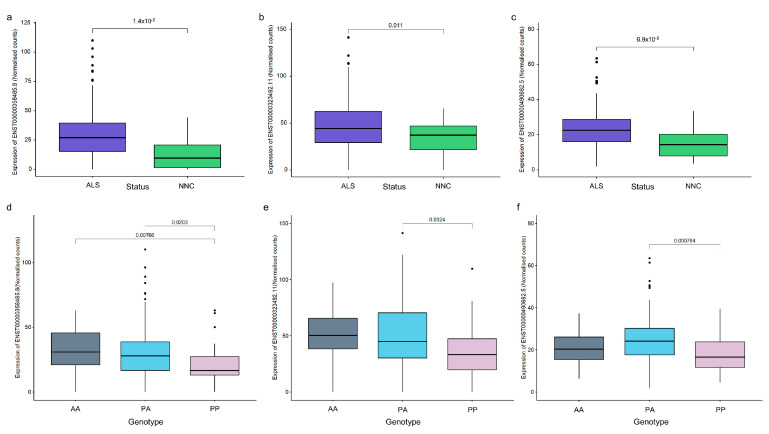
*CASP8* transcript expression is significantly higher in the spinal cords of individuals with ALS than NNC and is associated with SVA genotype. (**a**–**c**) Normalised counts of three *CASP8* transcripts significantly upregulated in the spinal cord (combined data from cervical, thoracic, and lumbar regions) of individuals with ALS or ALSND compared to NNC: ENST00000358485.8 (protein-coding), ENST00000323492.11 (protein-coding), and ENST00000490682.5 (non-coding). ALS n = 283 and NNC n = 31. Means were compared using a Wilcoxon test. (**d**–**f**) Normalised counts of three *CASP8* transcripts which showed a significant difference between at least two of the *CASP8* SVA genotypes in the spinal cords of individuals with ALS or ALSND. AA n = 41, PA n = 175, and PP n = 40. An ANOVA test with a Tukey adjustment for pairwise comparisons was performed. ALS–amyotrophic lateral sclerosis or ALS and other neurological disease; NNC–non-neurological control; AA–homozygous absent; PA–heterozygous for SVA insertion; and PP–homozygous present for SVA insertion.

The expression of *CASP8* annotated transcripts and the effects of the SVA variants were evaluated in the spinal cord transcriptome data from the Target ALS cohort as this tissue had the most robust *CASP8* expression. Four *CASP8* transcripts (ENST00000358485.8, ENST00000323492.11, ENST00000490682.5, and ENST00000673742.1) were expressed at a significantly higher level in the spinal cords of individuals with ALS or ALSND compared to the NNCs. Of these four transcripts, three showed differential expression between at least two *CASP8* SVA genotypes (Figure 5). The lowest expression was observed in individuals who were homozygous present for the SVA insertion.

### 2.4. Association Analysis of Polymorphic Reference SVAs with ALS and Their Functional Effects

Reference SVAs are known to be polymorphic for their presence/absence in the human genome. Therefore, these elements were genotyped in the ALS consortium dataset, and we used whole genome sequencing short-read data to investigate their potential role in the risk of disease. The ALS consortium dataset includes more than 4400 whole genomes from multiple different cohorts and individuals with a range of diagnoses including ALS and ALSND as well as non-neurological controls (NNC). In the 4416 individuals analysed, 95 reference SVAs were detected as absent in at least one individual. These 95 SVAs included 7 which were part of Gencode transcripts and 2 that were identified as novel exonised elements in the analysis described earlier in this study (highlighted in Supplementary Materials Tables S1 and S2, respectively). Due to the heterogeneity of the population within the dataset and the fact that SVA RIPs can be population-specific, association analysis was performed only on those identified as >90% European in the associated phenotype file (NNC = 322 and ALS/ALSND = 2663) (see Table 1 for demographic information). The 95 reference SVAs were then filtered to keep those with a minor allele frequency (MAF) above 5% in the European subset to undergo association analysis. In the resulting 65 SVA RIPs (MAF > 0.05) analysed, there were no elements associated with ALS/ALSND after correction for multiple testing (Supplementary Materials Table S3). Four SVA RIPs with an unadjusted *p* value < 0.05 (significant prior to correction) are shown in Table 2, which includes SVA_28 and SVA _82, which are located upstream of the Human Leukocyte Antigen A (*HLA-A*) and Zinc Finger Protein 780A (*ZNF780A*) genes, respectively, with one located in the 3′UTR of the Transferrin (*TF*) gene (SVA_20) and the other downstream of the Aquaporin 3 (*AQP3*) gene (SVA_51). In addition to the disease association analysis, we performed a linear regression analysis to determine if any of the SVA RIPs were associated with age at onset and survival analysis using the Cox proportional hazards model. After correction for multiple testing (Bonferroni correction), there were no SVA RIPs significantly associated with age at onset; however, the element with the lowest *p* value was again SVA_28, located upstream of the *HLA-A* gene. Individuals with ALS or ALSND had a lower average age at onset when the SVA upstream of *HLA-A* was present (AA—59.6 years, PA—58.5 years, PP—57.3 years, adj *p* = 0.097). There were also no SVA RIPs significantly associated with differences in survival in this cohort after correction for multiple testing (Bonferroni correction).

**Figure 6:**
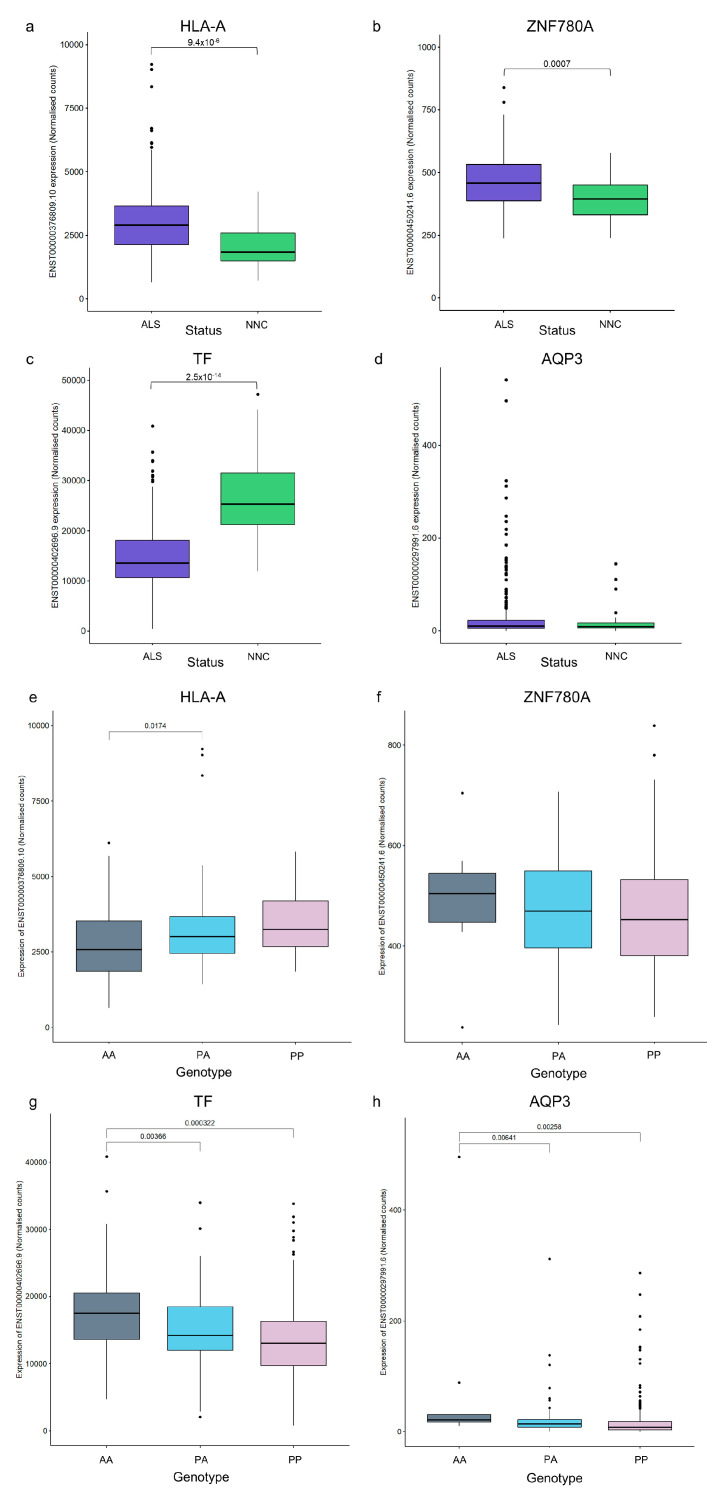
Transcripts proximal to polymorphic reference SVAs show a significant difference in the expression levels between individuals with ALS and controls in the spinal cord, and expression is influenced by the SVA genotype. Normalised counts of transcripts located proximal to the top four polymorphic SVAs identified in the disease association analysis are shown. (**a**) The expression of ENST00000376809.10 (*HLA-A* gene) was significantly higher in the spinal cords of individuals with ALS compared to NNC. (**b**) The expression of ENST00000450241.6 (*ZNF780A* gene) was significantly higher in the spinal cords of individuals with ALS compared to NNC. (**c**) The expression of ENST00000402696.9 (*TF* gene) was significantly lower in the spinal cords of individuals with ALS compared to NNC. (**d**) There was no significant difference in the expression of ENST00000297991.6 (*AQP3* gene) in the spinal cords of individuals with ALS compared to NNC. A Wilcoxon test was performed while considering Benjamini–Hochberg correction to compare the transcript levels between ALS and NNC for four transcripts in four CNS tissues: spinal cord, motor cortex, frontal cortex, and cerebellum (data from three brain regions shown in Supplementary Materials Figure S3). ALS n = 344 and NNC n = 39. (**e**) There was a significant difference in the expression of ENST00000376809.10 between the AA and PA genotypes of reference SVA_28 located upstream of the *HLA-A* gene (AA n = 181, PA n = 87 and PP n = 19). (**f**) There was no significant difference in the expression of ENST00000450241.6 between the different genotypes of reference SVA_82 located upstream of the *ZNF780A* gene (AA n = 10, PA n = 92 and PP n = 187). (**g**) There was a significant difference in the expression of ENST00000402696.9 between the AA and PA and the AA and PP genotypes of reference SVA_20 located in the 3′UTR of the *TF* gene (AA n = 25, PA n = 141 and PP n = 126). (**h**) There was a significant difference in the expression of ENST00000297991.6 between the AA and PA and the AA and PP genotypes of reference SVA_51 located downstream of the *AQP3* gene (AA n = 11, PA n = 81 and PP n = 199). An ANOVA test with a Tukey adjustment for pairwise comparisons was performed. ALS–amyotrophic lateral sclerosis or ALS and other neurological disease, NNC–non-neurological control, AA–homozygous absent, PA–heterozygous for SVA, and PP–homozygous present for SVA.

Although no SVA RIPs were significantly associated with ALS risk, age at onset, or survival after correction, we investigated the transcriptomic data from the Target ALS cohort. We evaluated the expression of the nearest gene to the SVA and the effects of the SVA RIP on its expression. This analysis revealed both the tissue- and disease-specific expression profiles of these gene transcripts (Supplementary Materials Figure S2, Figure 6 and Figure S3). The most highly expressed transcript of each gene was compared between individuals diagnosed with ALS or ALSND and NNCs, identifying a significant difference in the expression of the *HLA-A* (ENST00000376809.10), *ZNF780A* (ENST00000450241.6), and *TF* (ENST00000402696.9) gene transcripts but not in the expression of *AQP3* (ENST00000297991.6) in the spinal cord (Figure 6a–d). Expression levels were also compared in three brain regions (motor cortex, frontal cortex, and cerebellum). Only the *ZNF780A* transcript in the motor cortex and cerebellum displayed a significant difference in expression levels (Supplementary Materials Figure S3). Due to the differences in expression identified in the spinal cord, the effects of the SVA variants were evaluated in only those individuals with ALS or ALSND within that tissue. Individuals who were homozygous absent for the *HLA-A* SVA had significantly lower levels of *HLA-A* transcript expression than the heterozygous individuals (*p* = 0.0174) (Figure 6e). There were no significant effects regarding the genotype of the SVA upstream of *ZNF780A* on *ZNF780A* expression (Figure 6f). The expression of the *TF* transcript was significantly lower in PP and PA individuals compared to AA (*p* = 0.0003 and *p* = 0.0037) (Figure 6g). Finally, the expression of the *AQP3* transcript was significantly higher in AA individuals compared to PA and PP (*p* = 0.0064 and *p* = 0.0026) (Figure 6h).

## 3. Discussion

Using transcript databases and RNA sequencing data, we identified SVA sequences included in annotated transcripts and SVA exonisation events not previously characterised. In this study, we have demonstrated that the distinct expression profiles of these SVA-containing transcripts are dependent on tissue and disease status. These altered transcripts have the potential to encode novel proteins and affect cellular phenotypes. Some of the changes in expression observed involved genes in pathways previously implicated in ALS. Finally, SVA RIPs were characterised in the ALS consortium dataset, leading to the identification of elements whose genotype influenced transcript levels. This study has highlighted the functional effects of SVAs on mRNA splicing and expression in the CNS and the importance of assessing these elements in genetic and transcriptomic studies of neurological disorders such as ALS.

Our analysis of the Gencode database of transcripts with reference SVAs identified 179 transcripts containing sequences from 126 SVAs. This is higher than the number previously discovered using expressed sequence tags (ESTs); two separate studies identified 16 and 22 such events [18,22]. Of those identified, nearly two thirds accounted for non-coding transcripts, and the coding transcripts were predominantly located in the UTRs. There were 20 transcripts to which SVAs provided coding sequences; in 5 of these, the SVA introduced a stop codon prior to the last exon and underwent NMD. In the remaining 15 transcripts that did not undergo NMD (Figure 2 and Supplementary Materials Figure S1), the SVA formed the coding sequence of the first or last exon and the UTR. This suggests that SVAs located in the central introns of the gene are more likely to result in transcripts that are degraded than those located at the 5′ and 3′, and the latter group have the potential to contribute sequences to transcripts encoding new proteins. The transcripts partly encoded by SVAs include genes such as the leptin receptor involved in the regulation of body weight and a lysosomal acetyltransferase involved in the degradation of heparin sulphate. The expression levels of the SVA-containing transcripts were detected in multiple CNS tissues and differed, demonstrating that SVAs contribute to functional genes in a tissue-specific manner (Figure 2 and Supplementary Materials Figure S1).

SVA RIPs can be evaluated using whole genome sequencing data, identifying potential regulatory differences between individuals. We characterised SVA RIPs in the ALS consortium cohort from the New York Genome Center to evaluate their effects on gene expression and to identify any that might be involved in disease risk. ALS is a complex disease involving both genetic and environmental factors, and much of the heritability of the disease is yet to be determined. The missing heritability may partially lie in under-analysed complex variants such as SVAs [23,24]. Of the 126 SVAs within the Gencode transcripts, 7 were detected as RIPs in the ALS consortium. One of these elements was also one of the four SVAs with the lowest *p* value in the performed disease association analysis, although this result was not significant after correction for multiple testing (Table 2). This particular SVA (SVA_20) was located in the 3′UTR of the Transferrin (*TF*) gene. The TF protein is an iron transport protein; when comparing the expression levels of the *TF* transcript, there were significantly lower expression levels in the spinal cords of individuals with ALS/ALSND compared to the NNC group (Figure 6). Iron is involved in several important processes and helps to maintain normal physiological function in the CNS, which includes mitochondrial respiration and DNA and myelin and neurotransmitter synthesis; abnormal iron homeostasis can lead to oxidative stress and cellular damage [25]. Abnormalities in iron metabolism have been previously associated with ALS; a recent meta-analysis reported that serum TF levels were significantly reduced in ALS compared to healthy controls, whereas ferritin was significantly increased [26]. In addition, the level of the *TF* transcript containing the SVA was dependent on the SVA genotype, with lower levels of expression being associated with the presence of the SVA (Figure 6).

The three other SVAs with the lowest p values were not located in the exons of known transcripts but up- or downstream of the following genes: *HLA-A*, *ZNF780A*, and *AQP3*. SVA_28 located upstream of the *HLA-A* gene also had the lowest *p* value after correction (*p* = 0.097) in the age at onset analysis. There was a significantly higher level of expression of the *HLA-A* transcript in the spinal cords of individuals with ALS/ALSND compared to NNC. Furthermore, PA genotype individuals had a significantly higher expression than AA genotype individuals. We have previously shown that this particular SVA was associated with Parkinson’s disease progression and with *HLA-A* gene expression in the Parkinson’s Progression Markers Initiative (PPMI) cohort [7]. During the study of this SVA in the PPMI cohort, we identified variations in the size of this SVA as well as variations in the presence/absence. Variations in the sizes of SVAs have been associated with SNPs linked to neurodegenerative diseases, such as Alzheimer’s and Parkinson’s disease, and with differential gene expression [27]. Investigations into this additional layer of genetic variation regarding SVAs, in particular those highlighted in this study, in an ALS cohort could potentially further stratify those with the disease and characterise the SVA allele’s influence on gene expression.

Our study utilised RNA-sequencing data from the motor cortex to identify 92 SVA exonisation events which were not part of annotated transcripts. These 92 novel events occurred in both coding and non-coding transcripts, and six had been reported previously by Hancks et al. [18]. The majority of the novel exonisation events and the SVAs that are part of the annotated transcripts have been inserted into the same orientation as the gene (73% and 82%, respectively). This is significantly higher than the 29% of all intronic SVAs that are in the same orientation as the associated gene. We suggest that this may be due to the presence of multiple splice sites located in the sense orientation of the SVAs. Due to their potential functional impact on splicing patterns, this triggers selection against SVAs in the same orientation as the gene. SVAs have been shown to affect splicing patterns without being included in the mRNA sequence by altering splice site usage, and another type of retrotransposon, *Alu*, have been shown to alter mRNA splicing when located within 100 bp of exons. Therefore, we evaluated the exon–exon junctions adjacent to 27 reference SVAs located within 100 bp of an exon and identified two elements where adjacent exon–exon junctions were not part of the annotated transcripts. One of which was an SVA located in an intron of the *CASP8* gene and whose absence in the genome leads to the use of an alternative acceptor splice site 5′ of where the SVA would have been present; thus, a larger exon is included in the mRNA. Characterisation of the entire transcript sequence using long read RNA sequencing would be beneficial to understand the consequences of these SVA exonisation events. Using short-read sequencing to accurately determine the effects when multiple isoforms of a gene exist is difficult. In addition, our analysis could be extended to quantify the levels of the novel exonised SVAs and exon usage influenced by SVAs in those with and without ALS to determine if these changes are related to disease status. One of the hallmarks of ALS is TDP-43 dysfunction, a protein involved in regulating RNA splicing, and recent studies have shown that TDP-43 is involved in the suppression of cryptic exons [28,29,30,31]. It would be interesting to determine if SVA-associated splicing patterns are further regulated by TDP-43 and part of the exon usage changes observed in ALS.

Our study has highlighted the effects of reference SVAs on transcript splicing patterns and their expression in a tissue-specific manner. The increasing number of genomes sequenced and analysed using tools to detect retrotransposon variation is increasing the number of SVAs being discovered. However, it can be difficult to characterise the effects of variants in non-coding regions of the genome; therefore, combining both genomic and transcriptomic data can help in this endeavour. Understanding the functional consequences of these elements is important to determine the SVAs that impact normal cellular function and may be involved in disease development.

## 4. Materials and Methods

### 4.1. Identification of Gencode Transcripts Containing Exonised Reference SVAs

The coordinates of reference SVAs [32] were lifted over from hg19 to hg38 using the UCSC genome browser (https://genome.ucsc.edu/index.html, accessed on 24 January 2022). These were intersected with the coordinates of exons of Gencode transcripts (V39) in the UCSC table browser to generate a list of 126 SVAs whose sequence were part of 179 transcripts. Information regarding these transcripts was extracted to determine specific features such as whether they were coding or not.

### 4.2. DNA and RNA Samples from NIH NeuroBioBank

A total of 100mg of tissue from the motor cortices of 20 individuals with ALS and 20 without (unaffected individuals also had no significant neuropathology reported) was obtained from the NIH NeuroBioBank (https://neurobiobank.nih.gov/, accessed on 2 January 2019) through their application process. The tissue was divided into two; the DNA samples were extracted from one half using the Gentra Puregene Tissue kit (Qiagen, Hilden, Germany), and the RNA samples were extracted from the other half using the RNeasy Lipid Tissue mini kit (Qiagen, Hilden, Germany) following the manufacturer’s instructions. The extracted RNA underwent DNAse treatment using the Turbo DNA-free kit (Invitrogen, Waltham, MA, USA).

### 4.3. RNA Sequencing and Identification of Novel Exonised Reference SVAs

The RNA data extracted from 39 of the MCX tissue samples received from the NIH NeuroBioBank were sent to the Australian Genome Research Facility for library preparation (Stranded Total RNA with Ribo Zero Plus) and Illumina sequencing on the NovaSeq platform. The fastq files were aligned using STARv2.7.7a to generate bam files for visualisation in Integrative Genomic Viewer [33]. For the junction analysis, the fastq files were analysed using Rsubread with the *subjunc* function [34]. The junctions identified by the R Subread analysis were intersected with reference SVAs using the UCSC genome browser for 10 of the MCX samples analysed (5 ALS and 5 controls). To identify potential novel exonised SVAs, this list of junctions was manually inspected to identify those located in a reference SVA and neighbouring exon which were not an existing junction of the 179 SVA-containing transcripts previously identified.

### 4.4. Quantification of Transcript Expression in the Target ALS Cohort

Transcript-based analysis was performed using Salmon software (v1.10.1) [35]. Pair-ended fastq files were loaded, and counts were detected in a transcript-level resolution using human genome version 38 (GRCh38.p13).

### 4.5. Amplification of Novel Exonised SVA Sequences and Transcripts Using RT-PCR

Primers were designed and optimised to target three novel exonised SVA transcripts identified in the *UGGT2*, *MYO5A*, and *SLC25A12* genes identified in RNA sequencing data from the motor cortices of the individuals from the NIH NeuroBioBank cohort. Primers were also designed to evaluate the expression of *CASP8* transcripts and the effects of the polymorphic SVA within its intron. Primers were located in the exons flanking the SVA and/or within the SVA itself (See Supplementary Materials Table S4 for details). The targets were amplified using SuperScript III One-Step Rt-PCR System with Platinum Taq Polymerase (Invitrogen, Waltham, MA, USA) under standard conditions and 20 ng of RNA from selected samples from the NIH NeuroBioBank cohort as input.

### 4.6. Genotyping of SVA Located in CASP8 in the NIH NeuroBioBank Cohort

Three primers were designed for the amplification of the empty site and the 3′ junction of the insertion in separate reactions using GoTaq G2 Hot Start polymerase (Promega, Madison, WI, USA) under standard conditions (For 5′ AAGCCTGCAGAATCCAGCTA 3′, Rev 5′ ATCGTGGGGCTTGATCTCAA 3′, internal SVA primer 5′ TGTTTATCTGCTGACCTTCCC 3′). The resulting PCR products were analysed via agarose gel electrophoresis to genotype each sample for the presence or absence of insertion based on product size.

### 4.7. Genotyping of Polymorphic Reference SVAs and Disease Association Analysis in Whole Genome Sequencing Data

Whole genome sequencing data in cram file format aligned to hg38 were obtained from the New York Genome Center as part of the ALS consortium dataset, which includes samples from multiple different projects such as Target ALS and Answer ALS. The ALS consortium contains individuals with a range of diagnoses, such as ALS spectrum MND, other MND, other neurological disorder (including Parkinson’s disease and dementia), and ALS with other neurological disorder, as well as non-neurological controls (healthy controls). The structural variant caller Delly2 (https://github.com/dellytools/delly, installed 1 September 2022), with default settings, was used to identify structural variants in 244 individuals from the Target ALS cohort. The structural variants were merged from each individual, and deletions overlapping with reference SVAs were extracted. This generated a list of reference SVAs that were absent in at least one of the 244 individuals. This panel of reference SVAs sites was used in the second call step of Delly to generate genotypes for the entire ALS consortium cohort (4416 individuals). Focusing only on reference SVA variants in the larger set of samples reduced the computational resources required to genotype all of the genome-wide structural variants. The SVAs sites were filtered using VCFtools to keep only those with a PASS, and low-quality genotypes were also removed. There were 95 reference SVAs detected as absent in at least one of the 4416 individuals. Association analysis was performed on those individuals that were >90% European according to the ALS consortium metadata and those diagnosed with ALS spectrum MND or ALS spectrum MND with other neurological disorder (n = 2663) and compared to the non-neurological controls (n = 322) (see Table 1 for demographics). Association analysis of 65 polymorphic reference SVAs (minor allele frequency > 0.05) with ALS was performed using logistic regression—adjusted for age, sex, and sequencing preparation—in PLINK (v1.07), and *p* values were adjusted for multiple testing (Bonferroni correction). Age at onset analysis was performed using linear regression of age at onset on SVA genotypes with sex, sequencing platform, and site of onset as covariates; *p* values were adjusted for multiple testing (Bonferroni correction). Survival analysis was completed using the Cox proportional hazards model from the ‘coxme’ package in R, with sex, sequencing platform, age at onset, and site of onset as covariates; *p* values were adjusted for multiple testing (Bonferroni correction). Individuals in the ALS consortium dataset who were still alive were censored at their last follow-up.

## Figures and Tables

**Table 1:** Demographics of ALS consortium cohort.

	NNC (n = 322)	ALS/ALSND (n = 2663)
Gender		
Male	157 (48.8%)	1601 (60.1%)
Female	165 (51.2%)	1062 (39.9%)
Age * (years)		
Mean (min-max)	57.4 (17–90)	59.1 (12–90)

* For NNC age at collection (44 unknown) and ALS/ALSND age at symptom onset (159 unknown).

**Table 2:** Association analysis results of the four polymorphic reference SVAs that were significant prior to Bonferroni correction. P—present; A—absent.

Chr	Coordinates	ID	Minor Allele	MAF		OR (95% CI)	Unadj *p* Value	Bonferroni *p* Value
				NNC	ALS/ ALSND			
6	29,932,007–29,933,750	SVA_28	P	0.29	0.24	0.78 (0.65–0.94)	0.008	0.50
19	40,107,200–40,109,854	SVA_82	A	0.15	0.19	1.37 (1.07–1.75)	0.012	0.77
3	133,784,939–133,786,238	SVA_20	A	0.29	0.33	1.27 (1.04–1.55)	0.018	1
9	33,423,379–33,424,657	SVA_51	A	0.25	0.22	0.80 (0.65–0.97)	0.027	1

## Data Availability

The sequencing (RNA and WGS) data analysed in this study from the ALS consortium were obtained upon application to the New York Genome Center, and data requests can be made by completing a genetic data request form at ALSData@nygenome.org. Additional data from this study will be made available upon reasonable request.

## Supplementary Materials

The following supporting information can be downloaded at: https://www.mdpi.com/article/10.3390/ijms241411548/s1.
