# Supplementary material for: A Genome-Wide Screen for the Exonisation of Reference SINE-VNTR-Alus and Their Expression in CNS Tissues of Individuals with Amyotrophic Lateral Sclerosis
Source: Int J Mol Sci. 2023 Jul 17;24(14):11548. doi: 10.3390/ijms241411548 (PMC10380656; doi:10.3390/ijms241411548)
Supplement: Supplementary file 1 [file ijms-24-11548-s001.zip › Supplementary Figures Pfaff et al.pdf]

Supplementary Data

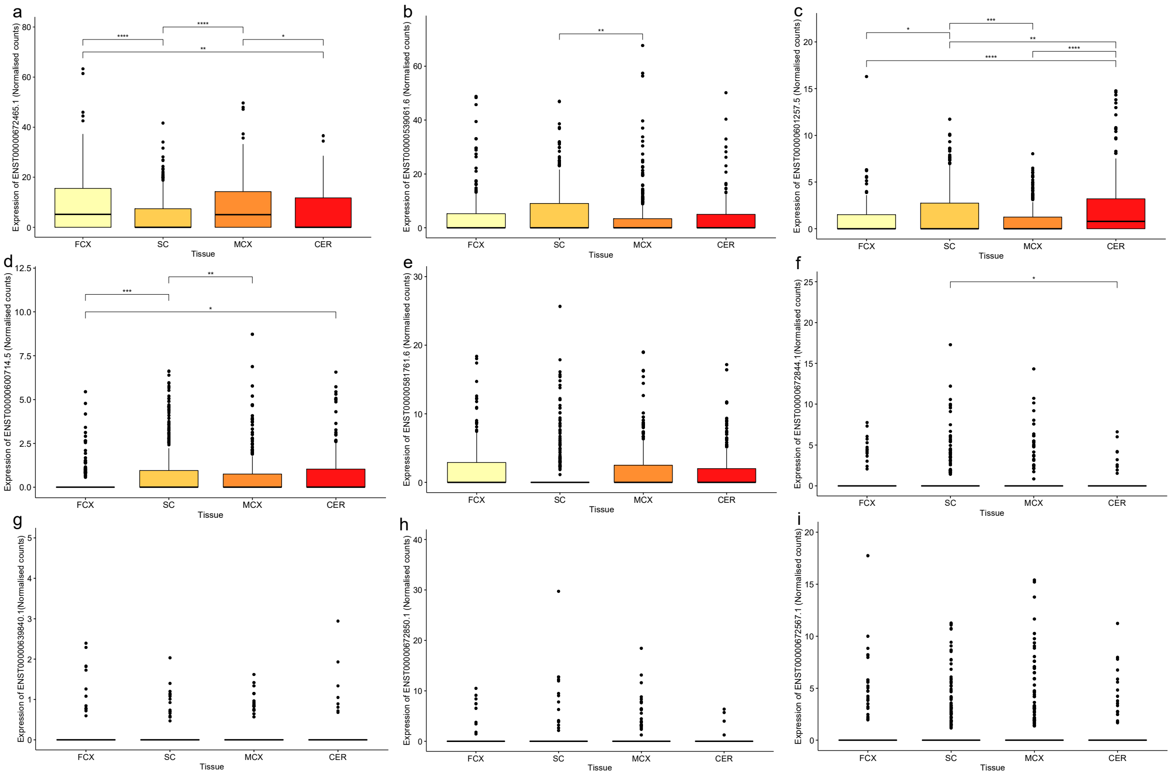

Figure S1: Expression of transcripts with coding exons that contain SVA sequences between tissues of the CNS. The graphs show normalised counts across four tissues from the Target ALS cohort for transcripts from the following genes: a) *ALDH3A2*, b) *C2CD3*, c) *ZNF83*, d) *ZNF83*, e) *MKS1*, f) *ANKDD1B*, g) *GLDC*, h) *ANKDD1B* and i) *ALDH3A2*. The number of samples analysed per tissue were as follows (includes data from both NNC and individuals with ALS or ALSND): FCX - 172, SC - 383, MCX – 317 and CER - 187. An ANOVA test with Tukey adjustment for pairwise comparisons was performed. *p<0.05, **p<0.01, ***p<0.001, ****p<0.0001. FCX - frontal cortex, SC - spinal cord (includes data from cervical, thoracic and lumbar regions), MCX - motor cortex (medial and lateral regions), and CER - cerebellum.

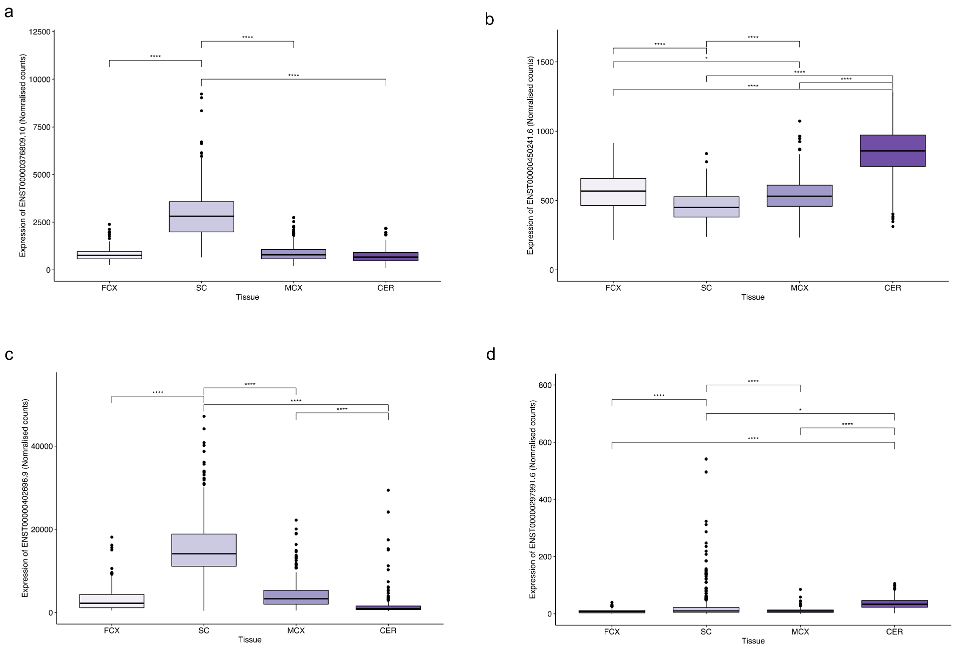

Figure S2: CNS tissue expression of transcripts located proximal to the top four polymorphic SVAs identified in the disease association analysis. There was a significant difference in the transcript expression between the tissues of the CNS for the four transcripts that were proximal to the top four SVAs from the association analysis (Table 2). Normalised transcript counts are shown for each tissue: a) ENST00000376809.10 (*HLA-A*), ENST00000450241.6 (*ZNF780A*), c) ENST00000402696.9 (*TF*) and d) ENST00000297991.6 (*AQP3*). The number of samples analysed per tissue were as follows (includes data from both NNC and individuals with ALS or ALSND): FCX - 172, SC - 383, MCX – 317 and CER - 187. An ANOVA test with Tukey adjustment for pairwise comparisons was performed. *p<0.05, **p<0.01, ***p<0.001, ****p<0.0001. FCX - frontal cortex, SC - spinal cord (includes data from cervical, thoracic and lumbar regions), MCX - motor cortex (medial and lateral regions) and CER - cerebellum.

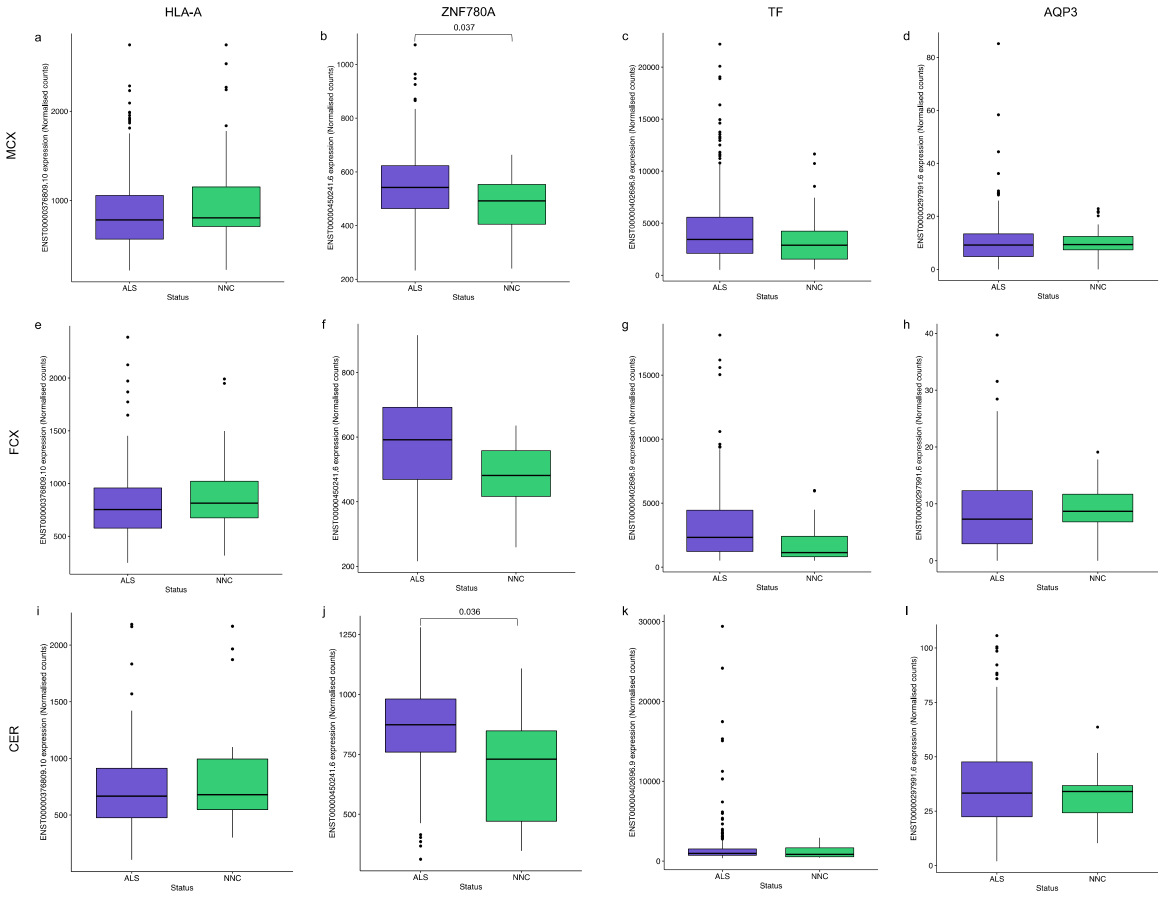

Figure S3: Expression levels of transcripts proximal to polymorphic reference SVAs in individuals with ALS and controls in three brain regions. Normalised counts are shown of transcripts located proximal to the top four polymorphic SVAs identified in the disease association analysis. a-d) In the motor cortex the expression of transcript ENST00000450241.6 (*ZNF780A*) was significantly higher in individuals with ALS or ALSND compared to NNC b) and there was no significant difference in the other three transcripts analysed: a) ENST00000376809.10 (*HLA-A*), c) ENST00000402696.9 (*TF*) and d) ENST00000297991.6 (*AQP3*). e-h) In the frontal cortex there was no significant difference in the expression of the four transcripts analysed between individuals with ALS or ALSND and NNC: e) ENST00000376809.10 (*HLA-A*), f) ENST00000450241.6 (*ZNF780A*), g) ENST00000402696.9 (*TF*) and h) ENST00000297991.6 (*AQP3*). i-l) In the cerebellum the expression of transcript ENST00000450241.6 (*ZNF780A*) was significantly higher in individuals with ALS or ALSND and NNC (j) and there was no significant difference in the other three transcripts analysed: i) ENST00000376809.10 (*HLA-A*), k) ENST00000402696.9 (*TF*) and l) ENST00000297991.6 (*AQP3*). Wilcoxon test was performed with Benjamini-Hochberg correction to compare transcript levels between ALS or ALSND and NNC for four transcripts in four CNS tissues: spinal cord, motor cortex (ALS n=280 and NNC n=37), frontal cortex (ALS n=153 and NNC n=19) and cerebellum (ALS n=170 and NNC n=17) (data from spinal cord is shown in figure 6a-d). FCX - frontal cortex, MCX - motor cortex (medial and lateral regions) and CER - cerebellum.
